# Nystagmus as an early ocular alteration in Machado-Joseph disease (MJD/SCA3)

**DOI:** 10.1186/1471-2377-14-17

**Published:** 2014-01-22

**Authors:** Mafalda Raposo, João Vasconcelos, Conceição Bettencourt, Teresa Kay, Paula Coutinho, Manuela Lima

**Affiliations:** 1 Center of Research in Natural Resources (CIRN), University of the Azores, Rua Mãe de Deus, Apartado 1422, 9501-801 Ponta Delgada, Portugal; 2 Institute for Molecular and Cellular Biology (IBMC), University of Porto, Porto, Portugal; 3 Department of Neurology, Hospital do Divino Espírito Santo, Ponta Delgada, Portugal; 4 Department of Molecular Neuroscience, UCL Institute of Neurology, London, UK; 5 Department of Clinical Genetics, Hospital of D. Estefania, Lisbon, Portugal; 6 CGPP, Institute for Molecular and Cellular Biology (IBMC), University of Porto, Porto, Portugal

**Keywords:** Spinocerebellar ataxia, Presymptomatic subjects, Clinical trials

## Abstract

**Background:**

Machado-Joseph disease (MJD), also named spinocerebellar ataxia type 3 (SCA3) is the most common autosomal dominant ataxia worldwide. Although nystagmus is one of the most frequently reported ocular alterations in MJD patients its behaviour during the course of the disease, namely in its early stages, has only recently started to be investigated. The main goal of this work was to characterize the frequency of nystagmus in symptomatic and presymptomatic carriers of the MJD mutation, and investigate its usefulness as an early indicator of the disease.

**Methods:**

We conducted an observational study of Azorean MJD family members, comprising a total of 158 subjects which underwent neurological evaluation. Sixty eight were clinically and molecularly diagnosed with MJD, 48 were confirmed asymptomatic carriers and 42 were confirmed non-carriers of the MJD mutation. The frequency of nystagmus was calculated for the 3 groups.

**Results:**

Nystagmus was present in 88% of the MJD patients. Seventeen percent of the at-risk subjects with a carrier result in the molecular test and none of the 42 individuals who received a non-carrier test result displayed nystagmus (p < 0.006). Although not reaching statistical significance, symptomatic subjects showing nystagmus had a tendency for a higher length of the CAG tract in the expanded allele, when compared to individuals who did not have nystagmus.

**Conclusions:**

The frequency of nystagmus in asymptomatic carriers and its absence in non-carriers of the mutation, suggests that nystagmus may appear before gait disturbance and can thus be considered an early sign of MJD.

## Background

Machado-Joseph disease (MJD; MIM #109150; ORPHA98757), also known as spinocerebellar ataxia type 3 (SCA3), is the most frequent autosomal dominant ataxia worldwide [1]. The MJD gene (*ATXN3*) was mapped to 14q32.1 and the causative mutation was identified as a coding unstable CAG expansion [2,3]; normal alleles consensually range from 12 to 44 CAGs, whereas well established limits for expanded alleles comprise from 61 to 87 repeats [4].

With an average onset in adulthood (around 40 years), MJD is characterized by a wide range of clinical manifestations, including ataxia, progressive external ophtalmoplegia, pyramidal and extrapyramidal signs, dystonia with rigidity and distal muscular atrophies [5,6]. Gait ataxia is reported as the initial complaint in the majority of MJD patients [7]; in some of the cases, other symptoms, such as diplopia have been reported as preceding gait instability [8]. Ocular alterations are clinical features commonly described in SCAs, occurring as a consequence of cerebellar and brainstem degeneration [9]. Amongst such alterations is nystagmus, a clinical sign associated with vestibular nuclei, found to be damaged in MJD post-mortem brains [10]. Nystagmus is one of the most frequently reported ocular alterations in MJD patients [7,11,12]; its behaviour during the course of the disease, namely in its early stages, has only recently started to be investigated [13].

The main goal of this work was to characterize the frequency of nystagmus in symptomatic and presymptomatic carriers of the MJD mutation and investigate its usefulness as an early indicator of the disease. The availability of molecular testing to detect asymptomatic carriers of the MJD mutation, associated with biological and/or refined clinical markers of the disease process, would enable an early intervention with prophylactic treatment, which should lead to prevention, or at least delayed onset or slowed progression, by allowing intervention before the appearance of motor symptoms [14].

## Methods

### Subjects

From 1996 to 2011, 158 subjects belonging to 21 Azorean MJD families performed the molecular test for MJD, either in the context of molecular diagnosis (N = 68) or predictive testing (PT) (N = 90). Subjects were asked for written informed consent to participate in research concerning the analysis of the clinical variability of MJD. During this period the molecular tests have been conducted by two distinct Portuguese genetic diagnosis laboratories.

Three main groups were defined for this study:

Group 1- subjects with a clinical and molecular diagnosis of MJD (N = 68). This group corresponds to individuals that were already symptomatic at the time of their first observation, and for which a molecular confirmation of MJD was subsequently obtained; Group 2- subjects with a normal neurological evaluation, who were confirmed as carriers of the *ATXN3* mutation in the molecular test (N = 48). Group 2 was therefore formed by subjects that were submitted to a neurological examination upon entering the PT program (before taking the molecular test) and were asymptomatic at that time. Some of the subjects from this group developed motor symptoms during the observational period of the study. For genotype-phenotype analysis these subjects were clustered with patients from group 1, constituting the pool of “symptomatic subjects” (N = 87).

Group 3- subjects with a normal pre-test clinical evaluation and that subsequently received a negative result in the molecular test (non-carriers of the *ATXN3* mutation) (N = 42).

This study is part of a larger project which was approved by the Ethics Committee of the Hospital do Divíno Espírito Santo (São Miguel Island, Azores – Portugal).

### Clinical features

Neurological examination was performed by two experienced neurologists, following the protocol established by Coutinho [7]. The examination included the evaluation of nystagmus, which consists in small, horizontal, saccade-like movements that lead the eye away from the target trajectory and, after a delay, bring it back onto the target [9,15]. The evaluation of nystagmus followed the ICARS procedure [16]. The age at onset and the duration of disease were also assessed. Onset corresponded to the age of appearance of the first symptoms (such as gait disturbance) reported by the patient and/or a close relative. Disease duration was calculated as the time elapsed between the age at onset and the last neurological evaluation available. Time from onset was calculated as the difference between age at first neurological observation and reported age at onset of ataxia.

### Statistical analysis

Differences in the frequency of nystagmus among the three study groups were measured by the Fisher’s Exact Test. Unrelated subjects (N = 46) from the pool of symptomatic subjects were analysed for the following variables: CAG length of the normal and expanded allele, age at onset and disease duration. Pearson correlation test was performed to evaluate the relationship between the CAG repeat length of normal and expanded alleles and age at onset. Univariate and multivariate linear regression were used to test the effect of several predictors on age at onset, namely: the number of CAGs in expanded and normal alleles, gender, island of origin, molecular diagnosis laboratory, and presence/absence of nystagmus. The best model was used to predict the age at onset for all members of group 2. The results were accepted as statistically significant whenever p < 0.05. All analyses were performed with PASW Statistics 18 [17].

## Results

### Frequency of nystagmus in symptomatic and presymptomatic MJD carriers

For the 158 Azorean subjects included in the study, information on gender, age and size of the CAG tract of MJD alleles is displayed in Table 1. Mean age is higher in group 1 (48 ± 15 years), than in the other 2 studied groups (30 ± 9 and 36 ± 12, respectively). The number of CAG repeats in the expanded allele is 71 ± 4 (mean ± Standard Deviation - SD) in both group 1 and group 2 (Table 1). Nystagmus was present in 88% of the MJD patients (group 1) and 17% of the at-risk subjects with a carrier result in the molecular test (group 2). However, nystagmus was not observed in any of the 42 individuals who received a non-carrier test result (group 3), which resulted in significant differences between groups 2 and 3 (p = 0.006).

**Table 1:** Characterization of the studied subjects according to group assignment

		**N**	**Group 1**	**N**	**Group 2**	**N**	**Group 3**	
	**Gender**	68	33♀|35♂	48	29♀|19♂	42	26♀|17♂	
	**Age**	68	48 ± 15 [16–82]	48	30 ± 9 [18–59]	42	36 ± 12 [18–60]	
**CAG repeats**	Normal allele	60	20 ± 5 [13–28]	44	22 ± 5 [13–27]	42	17 ± 4 [13–27]	23 ± 4 [13–31]
	Expanded allele	64	71 ± 4 [61–80]	46	71 ± 4 [63–80]			

All variables were presented as mean ± standard deviation [range]. Gender was represented as ♀ for female and ♂ for male.

From the 68 patients in group 1, only eight (12%) failed to present nystagmus at the first neurological examination; five out of these eight, however, presented nystagmus at the last observation performed in the course of this study, increasing to 96% the number of patients who presented nystagmus. The remaining three patients were lost from follow-up, therefore preventing the verification of the presence of nystagmus at a later stage of the disease.

**Figure 1:**
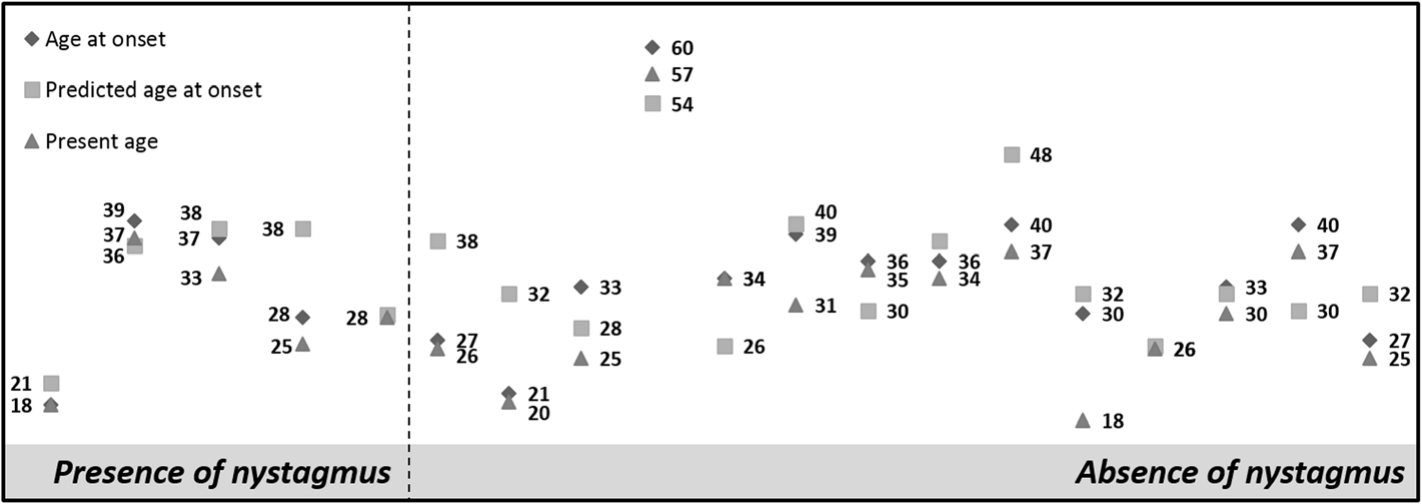
Age at onset, predicted age at onset and present age was displayed in individuals from group 2, which during this study developed the disease, divided by presence (N = 5) and absence of nystagmus (N = 14).

From the group of 48 asymptomatic carriers (group 2), 19 (40%) developed gait disturbance within the period of this study. From these 19, 5 already presented nystagmus at PT evaluation and developed gait ataxia during the follow-up period; the remaining 3 were not re-evaluated (Figure 1). Fourteen individuals from group 2 also developed gait ataxia during this period (Figure 1); for such subjects nystagmus, although absent in the respective PT evaluation, was present at the latest observation performed. Individuals which presents nystagmus at PT evaluation were closest to reported age at onset (median of 2 years) than individuals without nystagmus (median of 3 years).

### Genotype-phenotype correlations

**Figure 2:**
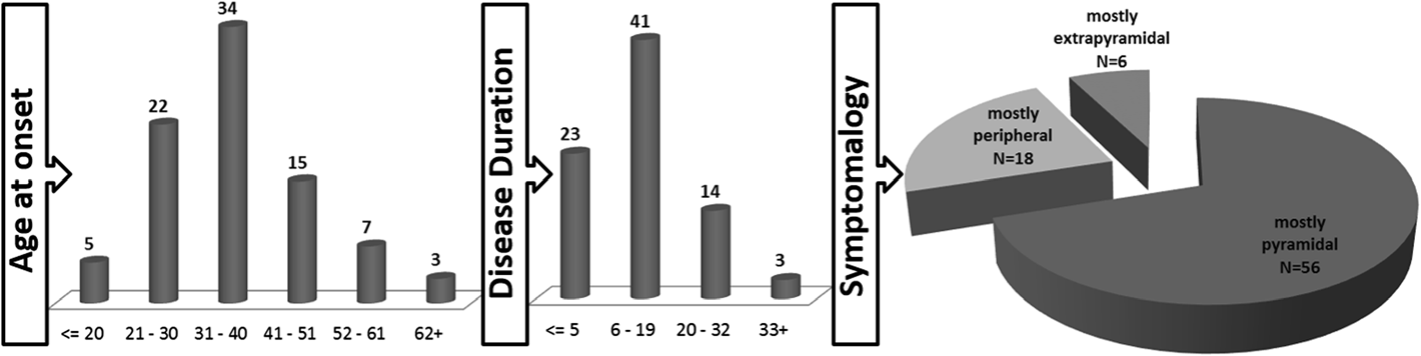
**Neurological features of MJD patients, such as age at onset (N = 86), disease duration (N = 81) and symptomatology (N = 68).** Cerebellar dysfunction was observed in all patients and therefore they were distinguished also by dysfunction in other systems: mostly corticospinal, mostly extrapyramidal or mostly peripheral signs.

The 87 symptomatic carriers were clinically characterized, showing a mean value for age at onset of 39 ± 12 years (mean ± standard deviation) and disease duration of 15 ± 9 years. All subjects displayed cerebellar signs; moreover, 65% presented mostly corticospinal dysfunction, followed by mostly peripheral (26%) and extrapyramidal (6%) manifestations (Figure 2).

Using information on unrelated symptomatic subjects with available CAG data, a negative correlation between the size of the expanded allele and the age at onset was observed (N = 42, r = -0.667, p < 0.0005). The explanation of the age at onset variance provided by the CAG length in expanded allele was 45% (t = -5.740, p < 0.0005). An improvement of the model using additional predictors (CAG repeat number in normal allele, gender, island of origin and genotyping laboratory) raised the explanation of the age at onset to 48% (p < 0.0005). Individuals showing nystagmus had a tendency for a higher CAG length in expanded allele, when compared to individuals who did not have nystagmus [71.54 ± 0.67 and 69.71 ± 0.97 (mean ± standard error), respectively]; this tendency, however, failed to reach significance. The presence/absence of nystagmus did not improve significantly the prediction of the age at onset when adding this variable to the number of CAG repeats in the expanded allele as well as the other predictors previously mentioned.

## Discussion

The present study confirmed nystagmus as a very frequent sign in MJD patients (up to 96% of the MJD patients from the studied series), which is in agreement with previous studies where this sign has been reported with frequencies that vary between 55 and 92% [7,8,18,19]. Considering the genotype-phenotype results, a long term follow-up study would be needed to understand if there is a correlation between CAG repeat length in expanded allele and the age of appearance of nystagmus.

The present study further shows that nystagmus is also observed in carriers of the MJD mutation, before the manifestation of gait disturbance, indicating that it can be an early sign of the disease, which should be monitored in mutation carriers even before motor signs appear. Recently a higher rate of horizontal gaze-evoked nystagmus in MJD carriers than non-carriers (ten [39%] of 26 vs one [5%] of 20; p = 0 · 013) has been described [13]. From ataxia clinical scales currently being applied, the complementary use of one that includes the evaluation of nystagmus, such as ICARS [16] or NESSCA [20] is important, to avoid the under-estimation of this clinical sign and the failure to detect early alterations. In other SCAs, early detection of impaired eye movements in presymptomatic individuals has been a matter of study. Slowing of horizontal saccades was observed in SCA1, 2, 6 and 7 [19,21-24] whereas a decrease in pursuit gain was found in SCA6 [25]. The present study is based in the qualitative analysis of nystagmus, i.e., presence or absence of this clinical sign; although reasonable information about oculomotor movements might be obtained with such qualitative measure, the use of semi-quantitative techniques such as oculographic evaluations, should improve the characterization of nystagmus types as well as the study of other ocular features, which might help to further understand the importance of eye abnormalities in early stages of MJD.

## Conclusion

The presence of nystagmus in asymptomatic MJD carriers suggests that the detection of this sign might be useful in the identification of early stages of MJD, potentially facilitating the enrolment of presymptomatic individuals in future preventive clinical trials.

## Competing interests

The authors declare that they have no competing interests.

## Authors’ contributions

MR and ML participated in the design of the study and drafted the manuscript. JV and TK were involved in the collection of the clinical data. CB and PC participated in the discussion of the study and drafted the manuscript. JV and ML participated in coordination of the study. All authors read and approved the final manuscript.

## Pre-publication history

The pre-publication history for this paper can be accessed here:

http://www.biomedcentral.com/1471-2377/14/17/prepub
